# Congenital Insensitivity to Pain (HSNA type IV)

**DOI:** 10.15844/pedneurbriefs-29-4-6

**Published:** 2015-04

**Authors:** J. Gordon Millichap

**Affiliations:** 1 Division of Neurology, Ann & Robert H. Lurie Children's Hospital of Chicago, Chicago, IL; 2 Departments of Pediatrics and Neurology, Northwestern University Feinberg School of Medicine, Chicago, IL

**Keywords:** Congenital, Pain, Norepinephrine, Anhidrosis

## Abstract

Investigators from New York University, NY, studied 14 patients with congenital insensitivity to pain with anhidrosis (CIPA), compared to 10 patients with chronically deficient sympathetic activity (pure autonomic failure), and 15 normal age-matched controls.

Investigators from New York University, NY, studied 14 patients with congenital insensitivity to pain with anhidrosis (CIPA), compared to 10 patients with chronically deficient sympathetic activity (pure autonomic failure), and 15 normal age-matched controls. In all patients with CIPA, plasma norepinephrine levels were very low or undetectable and failed to increase in the upright posture despite normal blood pressure. Plasma epinephrine levels were normal and increased when patient was upright. Plasma renin activity also increased appropriately with upright posture. Patients with pure autonomic failure also had very low levels of plasma norepinephrine both supine and when upright, but in contrast to patients with CIPA failed to maintain blood pressure upright.

In CIPA, postganglionic sympathetic neurons are severely depleted, but adrenal medulla chromaffin cells are spared. It is postulated that in the congenital absence of norepinephrine, adrenal epinephrine “takes over” sympathetic neurovascular signaling to increase peripheral vascular resistance and maintain blood pressure. CIPA is inherited as autosomal recessive with mutations in the gene TRKA forming part of the receptor for nerve growth factor (NGF). In skin biopsies of patients with CIPA sweat glands are preserved but lack sympathetic cholinergic innervation, explaining the anhidrosis. [1]

COMMENTARY. Congenital insensitivity to pain with anhidrosis (CIPA), also known as hereditary sensory and autonomic neuropathy (HSAN) type IV, presents in infancy with oral self-mutilation and burns, sometimes mistaken for child abuse [2]. Other features include multiple bone fractures and musculoskeletal complications, scars, osteomyelitis, joint deformities, ocular complications, and hyperpyrexia. Mental retardation and behavior problems are common. Although considered rare, in Japan this disorder has warranted the formation of a Research Group on CIPA and HSAN types IV and V, with publication of Guidelines of Total Management and Care for CIP [3]. Both types IV and V are characterized by CIP whereas type IV is also complicated by anhidrosis and intellectual disability.

The neurotrophin nerve growth factor (NGF) is essential for the development of sensory neurons and pain perception. A homozygous missense mutation in the NGF gene has been identified in HSAN IV and V patients. Genetic studies of these disorders should unravel the mechanism of CIP [4]. In a reference cited by Adams and Victor, the autopsy of a patient who died at age 12 years showed absence of small neurons in dorsal root ganglia, absence of Lissauer tracts and descending spinal tracts of trigeminal nerves, and preserved skin sweat glands but lacking innervation [5].
